# Modeling of the Impact of the Credibility of the Destination Endorser on the Place Attachment of Potential Tourists

**DOI:** 10.3389/fpsyg.2021.759207

**Published:** 2021-11-26

**Authors:** Ziye Shang, Jian Ming Luo

**Affiliations:** Faculty of International Tourism and Management, City University of Macau, Macao, Macau SAR, China

**Keywords:** place attachment, endorser credibility, destination attitude, para-social relationship, destination source credibility

## Abstract

Place attachment has been identified as effective marketing strength to enhance destination competitiveness. However, little research explored the formation mechanism of the place attachment of potential tourists and the role of celebrity endorsers. This study aims to identify the influence mechanism of the credibility of endorsers on place attachment. Various theories from different research fields were introduced to explore the mediating effect of destination attitude, para-social relationships, and destination source credibility between endorser credibility and the place attachment of potential tourists. The famous destination endorser in China, Tenzin, was chosen as the case. A quantitative method was adopted. The research model was examined by a survey sampling size of 367 respondents. The covariance-based structural equation model analysis revealed that the credibility of the endorser exerts a positive influence on the destination attitude of potential tourists, para-social relationship with the endorser, and destination source credibility. These three constructs have a positive effect on their place attachment. The results showed that the credibility of the endorser has a significant indirect influence on place attachment. This study extends the current literature of forming the place attachment of potential tourists from the perspective of the personal characteristics of the destination endorser. The findings shed light on how the credibility of the endorser could form place attachment through enhancing the destination attitude, credibility, and para-social relationships of potential tourists. This study provides several practical implications for destination marketing.

## Introduction

The increasing competition of destinations leads to the homogeneity of both tangible and intangible attributes among them (Van der Veen and Song, 2014; Zhang et al., 2020). Facing such a marketing context, place attachment, one of the main concepts in environmental psychology, has attracted the wide attention of tourism research (Dwyer et al., 2019). From the tourist perspective, it refers to the positive bond between tourist and destination (Tsai, 2012), which reflects the multiple meanings the tourists ascribe to the destination (Dwyer et al., 2019). Considering the critical role of place attachment on the behavior intention of tourists, revisit intention (Majeed and Ramkissoon, 2020), place satisfaction (Ramkissoon et al., 2013), and destination loyalty (Dwyer et al., 2019), prior studies deemed forming the place attachment of tourists as a distinctive tourism marketing strength to enhance the destination competitiveness (Dwyer et al., 2019). Many prior researchers postulated the individual place attachment derived from long-term experience with the environment (Woosnam et al., 2018; Vada et al., 2019). Factors such as the attractiveness of the destination, experience of tourists, motivation, and service interactions have been identified as antecedents of the place attachment of tourists (Dwyer et al., 2019). Corresponding to this post-visit perspective, another stream of studies proposed that place attachment could be fostered without enduring experience and even before the individual actually arrives at the destination. Few studies started to focus on the antecedents of the place attachment of potential tourists. The role of narrative transportation of song or film related to a destination as well as the physical elements of destinations familiarized by tourists has been identified as antecedents that could foster the place attachment of potential tourists (Cheng and Kuo, 2015; Chen, 2018; Chen et al., 2019; Hosany et al., 2020).

Compared with the extensive research on the place attachment of prior tourists, a need remains to further explore the formation mechanism of the place attachment potential tourists (Cheng and Kuo, 2015). The celebrity endorser is an effective way for destination marketing to capture the attention of potential tourists and act as a differentiator (van der Veen, 2008). Prior studies revealed that the celebrity endorser would be more effective in forming the attitude of audiences and facilitating their visit intention than generic advertisements (Roy et al., 2021). Although the role of the attributes or personality of celebrity endorsers on destination image and visit intention has attracted extensive scholarly attention (Lee et al., 2008; Van der Veen and Song, 2014; Yen and Teng, 2015; Xu and Pratt, 2018; Kim et al., 2019), its influence on generating the place attachment of potential tourists are relatively lacking (Chen, 2018). More specifically, the persuasive role of the credibility of endorsers has been identified in the marketing field and destination marketing context (van der Veen, 2008; Van der Veen and Song, 2014). However, the advantage of a credible endorser on forming the place attachment of potential tourists requires further research.

This study aims to explore the mechanism of a credible destination endorser to forming the place attachment of potential tourists. Attitude and personal relationships are significant concepts in social psychology (Gawronski, 2007; Thibaut and Kelley, 2017). The objectives of this study are (1) to explore the relationship between the credibility and place attachment of a celebrity endorser; (2) to verify the mediation role of destination attitude of potential tourists, para-social relationships and destination source credibility in this relationship; and (3) to offer some practical implications for destination managers. Combined with prior empirical findings, trust transfer theory (Strub and Priest, 1976), uncertainty reduction theory (Berger and Calabrese, 1974), and source credibility model (Hovland and Weiss, 1951) are introduced to establish the theoretical model. From these theoretical perspectives, the credibility of the endorser may change the attitude of potential tourists toward a destination, foster their para-social relationships and enhance their perception of destination source credibility, which in turn formats place attachment before they actually arrive.

The significance of this study is threefold: Firstly, prior studies mainly focused on the formation of place attachment after tourists visit the destination (Dwyer et al., 2019), few studies have investigated the formation mechanism of the place attachment of potential tourists. Secondly, the role of the characteristics of destination endorsers on forming place attachment received little academic attention, this study would extend the literature of this realm. Thirdly, this study combines various theories: trust transfer theory, uncertainty reduction theory, and source credibility theory to attempt to provide a comprehensive view of the formation mechanism of tourists. Overall, this study would advance our understanding of the role of destination endorsers on the place attachment of potential tourists by exploring the influence mechanism of the credibility of endorsers and the mediation role of the attitude, destination credibility, and para-social relationship of tourists.

## Literature Review

### Celebrity Endorser in Destination Marketing

Celebrity endorser refers to ‘the individual who enjoys public recognition and who uses this recognition on behalf of a consumer good by appearing with it in an advertisement’ (McCracken, 1989). The media personas such as pop stars, politicians, athletes, or social CEOs are traditional celebrities. Following the definition, the individual who becomes famous on social media platforms can also be defined as a celebrity in the web 2.0 age (Yang, 2018).

In the field of tourism, previous studies explored the role of destination endorsers in destination marketing from two perspectives. One perspective views the attributes of the endorser. Roy et al. (2021) revealed the possible impact of celebrity country of origin on the attitude and intention of tourists. Glover (2009) argued that the celebrity image may influence the destination awareness and decision making of tourists. Xu and Pratt (2018) explored the relationship among endorser–destination congruence, endorser–tourist congruence, attitude toward a destination and visit intention. Schoner-Schatz et al. (2021) verified that a smiling endorser in social media content more likely evoke the visit intention of tourists and their recommendation compared with non-smiling endorsers. In addition, some studies focus on the effect of the credibility of the destination endorser on the attitude toward a destination, destination image, awareness, perceived quality, brand passion, or brand love (Van der Veen and Song, 2014; Kim et al., 2018; Gilal et al., 2020; Kim and Chen, 2020; Zhang et al., 2020). The second perspective focuses on the characteristics of tourists. McCartney and Pinto (2014) revealed that the effect of destination endorsers on the decision making of tourists may vary with the demographic characteristics of tourists. Roy et al. (2021) found that the country of origin of tourists may influence the effect of the endorser on their decision making. Glover (2009) believed that the self-image of tourists may influence their destination awareness and decision making. Yen and Teng (2015) found that the celebrity involvement of tourists positively influences their perceived value of film scene and visit intention. Combined with para-social interaction theory and balance theory, Su et al. (2011) found that the para-social relationship of audiences with celebrities is positively related to their attitude toward a destination.

Despite the extensive investigation on the role of endorsers, few studies have explored their effect on place attachment. Kim et al. (2018) showed how the local celebrities of a destination influence the place attachment of tourists by enhancing brand awareness, perceived quality, and brand image. Chen (2018) found that the involvement of tourists with celebrities positively influences their destination image and their place attachment. However, the formation of the place attachment of potential tourists continues to lack attention. Zhang et al. (2020) explored how the credibility of endorsers and the para-social relationships of tourists influence tourist destination brand love. In this study, we explore how the credibility of celebrity endorsers influences the place attachment of potential tourists by enhancing the destination attitude, para-social relationship, and destination source credibility of potential tourists.

### Endorser Credibility and Destination Source Credibility

Credibility refers to the believability of information and/or its source (Hovland et al., 1953). In tourism research, prior studies explored the source credibility of online information (Xie et al., 2011; Ayeh et al., 2013; Ayeh, 2015; Tan and Chang, 2016; Dedeoglu, 2019), destination endorser, or the credibility of celebrities (Kim et al., 2018; Kim and Chen, 2020; Zhang et al., 2020), as well as destination source credibility (Veasna et al., 2013; Girish et al., 2020; Jiménez-Barreto et al., 2020). In this study, we focus on the latter two concepts.

Endorser credibility refers to the extent to which the endorser is perceived as possessing expertise relevant to the communication topic and can be trusted to provide an objective opinion on the subject (Goldsmith et al., 2000). In other words, credibility reflects the influence of the positive characteristics of endorsers on the acceptance of relevant information of the receivers (Ohanian, 1990). Physical attractiveness, trustworthiness, and expertise are the three basic sub-dimensions of credibility identified in previous studies. Physical attractiveness can be defined as the tendency or predisposition of the receiver to evaluate the physical attributes of endorsers in a positive way (Berscheid and Hatfield, 1969). Trustworthiness reflects the level of honesty and integrity of endorsers. Expertise refers to the extent to which an endorser is perceived to have competence and knowledge (McGinnies and Ward, 1980). Previous studies introduced various theories to explain the role of the credibility of endorsers on destination marketing. From the source credibility model perspectives, Van der Veen and Song (2014) explored how the perceived credibility of the endorser in print advertisements influences the destination attitude of receivers and the indirect effect on visit intention. Based on balance theory and direct effect transfer model, Zhang et al. (2020) constructed the relationships among the credibility of celebrities, the para-social relationships of tourists with celebrities, and their destination brand love. The positive impact of credibility on destination brand love was tested. In the lens of symbolic communication theory, Kim et al. (2018) verified the relationships among the credibility of endorsers, festival brand awareness, perceived quality, and festival brand image. By introducing the meaning transfer model, Kim and Chen (2020) explored the influence of the credibility of religious celebrities on the destination image and attachment of visitors. Gilal et al. (2020) verified the positive role of endorsers on brand passion.

Destination source credibility can be defined as the extent to which an individual believes the willingness and capability of destination management to deliver its promises related to the destination (Veasna et al., 2013). This concept is based on brand credibility derived from the brand signaling theory (Erdem and Swait, 1998). Prior studies in the marketing field believed that brand credibility is vital in brand choice under the asymmetric information of markets (Erdem and Swait, 2004). In tourism research, few studies explored the impact of destination source credibility. Veasna et al. (2013) found that higher destination source credibility could enhance the destination image of tourists and their place attachment. del Barrio-Garcia and Prados-Peña (2019) verified the positive effect of destination brand extension credibility on the perception of brand equity of tourists. From a pre-visit perspective, previous research found that online destination brand credibility may positively influence the behavioral intention of users toward the destination. Given the important impact of destination source credibility, prior studies also identified some of its antecedents. In a heritage tourism context, del Barrio-Garcia and Prados-Peña (2019) explored how the destination brand extension authenticity influences credibility. Focused on an online destination, Jiménez-Barreto et al. (2020) verified the positive influence of online experience on online destination credibility. To the best of our knowledge, the influence of the credibility of the destination endorser on destination source credibility remains unknown.

### Destination Attitude

The concept of attitude refers to an overall evaluation of a psychological object (Ajzen, 2001). The attitude of tourists toward a destination reflects their level of favorable evaluation or appraisal of a certain destination (Ajzen, 1991; Jalilvand et al., 2012). Tourist attitude has been recognized as a crucial factor of travel decisions of tourists (Jalilvand and Samiei, 2012; Shin et al., 2022). In terms of the determinants of tourist attitude, previous studies explored the tourist and destination perspectives. For example, the motivation (Pereira et al., 2019), beliefs (Lam and Hsu, 2006) and destination image of tourists; the personality of the destination (Souiden et al., 2017); and perceived value (Lee et al., 2014) have been verified as a predictor of tourist attitude. Apart from the two perspectives, some studies also identified the role of other sources. For instance, the attitude of users toward websites (Tang et al., 2012), electronic word of mouth (Jalilvand and Samiei, 2012), and source credibility (Van der Veen and Song, 2014) have been proven to be a predictor of tourist attitude toward a destination.

### Para-Social Relationships

The concept of para-social relationships (PSR) is derived from para-social interaction theory, which proposes that the media audience could develop an imagined intimacy with the media persona through one-sided interaction controlled by the media persona (Horton and Richard Wohl, 1956). The initial focus of this theory is the persona in mass media, such as newscaster (Rubin and McHugh, 1987), performer (Turner, 1993) on television, or host (Rubin and Step, 2000) on the radio. The theory postulates that the viewer would generate an illusion of social interaction through the appearance of the media persona, gestures, and voice communicated by media (Horton and Richard Wohl, 1956). In the age of web 1.0, the theory further developed in the context of website media. Hoerner (1999) proposed that corresponding to the social cues of the media persona, the website could also elicit an illusion of social interaction through the tone of narrative text, picture presentations, or design metaphor. The theory is then wildly introduced in the context of web 2.0. The role of PSR with persona was identified in various social media types, such as blogger (Thorson and Rodgers, 2006), vlogger (Hwang and Zhang, 2018), brand page (Labrecque, 2014), and online community (Zheng et al., 2020).

In the field of tourism, previous studies explored the role of PSR from various perspectives. Focusing on the benefits and behavior of tourists, Kim and Kim (2017) explored how the PSR of elderly users with tourism websites affects their well-being and word-of-mouth (WOM). Choi et al. (2019) explained the influence of the PSR of online travel community users on their community satisfaction and travel satisfaction. Yılmazdoğan et al. (2021) revealed that the PSR of viewers with social media influencers may influence their travel intention. From tourism company branding perspectives, Lee and Lee (2017) tested the relationships between the PSR of users with hotel brand through the social media platform and their self-brand connection and brand usage intention. Similarly, previous studies have identified the roles of PSR on tourism company brand identification and the citizenship behavior of consumers (Kim and Kim, 2018; Ye et al., 2021). From destination marketing perspectives, Su et al. (2011) found that the PSR of viewers with TV characters and their attitude toward the characters are related to destination attitude in a highly perceived cultural proximity context based on balance theory. Similarly, based on balance theory, Zhang et al. (2020) revealed that the PSR of viewers with a celebrity may positively influence their destination brand love.

### Place Attachment

The concept of place attachment derived from attachment theory initially focused on emotional bonding between humans (Bowlby, 1969). In environmental psychology, place attachment refers to the emotional bonding between individuals and places (Low and Altman, 1992; Hidalgo and Hernandez, 2001). Some studies proposed that the long-term experience of an individual of the physical and social aspects of a place (Ramkissoon et al., 2018; Ramkissoon, 2020), such as biology, environment, psychology, and sociocultural context of the place, develops their place attachment (Low and Altman, 1992; Stylidis, 2018; Woosnam et al., 2018). However, another line of studies argued that individuals could develop an attachment toward a place before they arrive there (Feldman, 1990; Blake, 2002), which emphasizes the role of the secondary source on place attachment (Beckley et al., 2007).

In tourism research, most studies explored the place attachment of residents (Strzelecka et al., 2017; Ramkissoon, 2020), tourists (Jiang et al., 2017; Aleshinloye et al., 2020), or employees (Stylidis, 2020) in the post-experience context. Research scarcely focuses on this concept in the pre-visit context. Chen et al. (2019) revealed that the listener may develop destination attachment through transportation of songs related to the destination. Cheng and Kuo (2015) found that the familiarity of destination elements for first-visit tourists has a positive relationship with their destination attachment. For destination marketing, Chen (2018) explored the positive influence of the celebrity involvement of audiences on their place attachment. Hosany et al. (2020) proposed that the potential tourist could develop a destination attachment through media persona.

### Hypothesis Development

#### Endorser Credibility and Destination Attitude

The source credibility model (Hovland and Weiss, 1951) is one of the celebrity endorsement strategy models proposed by previous marketing research. This theory postulates that the credibility of endorsers could influence the beliefs, attitudes, and behaviors of receivers toward the endorsed objects. The mechanism underlying this process is the internalization of the receivers, which means the endorser-induced source is congruent with the value structure of the receivers and thus has intrinsic rewards for them (Kelman, 1958; Erdogan, 1999). Therefore, the higher the credibility of the destination endorser, the more positive the attitude of the receiver toward the destination.

In the marketing field, earlier research has verified this hypothesis relationship. For example, La Ferle and Choi (2005) found that the credibility of celebrity endorsers could positively influence the attitude of consumers toward the product under experimental research. In the field of tourism, little research empirically verified this relationship. Van der Veen and Song (2014) tested the relationship between attractiveness, believability (i.e., trustworthiness and expertise) of print advertisement and the attitude of tourists toward destinations. The results showed that only the source attractiveness has a significantly positive influence on this attitude. Wang et al. (2017) found that the credibility of endorsers has a positive influence on the airline brand attitude of consumers. Similarly, Wang and Scheinbaum (2018) revealed that the attractiveness and trustworthiness of endorsers positively influence the attitude of passengers toward airline brands. Based on the above theory and empirical evidence, we proposed that

H1: The credibility of the destination endorser has a positive influence on the attitudes of potential tourists toward a destination.

#### Endorser Credibility and Para-Social Relationships

Previous studies have found contrary findings on the relationship between endorser credibility and PSR. Some studies argued that the para-social relationships may positively influence endorser/source credibility (Reinikainen et al., 2020). By contrast, other studies verified a significant influence of credibility on para-social relationships (Yuan et al., 2016). This study explains this relationship from the uncertainty reduction theory (URT) (Berger and Calabrese, 1974) perspective. The URT could support the positive influence of endorser credibility on PSR. According to this theory, the development of interpersonal relationships goes through three stages, and the initial stage is crucial in this process. The theory postulates that the primary concern in the initial stage is to reduce the uncertainty and thus enhance the predictability between two strangers. The lower levels of uncertainty toward others, the higher levels of liking between communicators. As mentioned above, credibility reflects the extent to which the receiver perceived that the endorser can be trusted and possesses the expertise on the relevant topic. Therefore, the higher the level of credibility of the endorser, the lower the level of the uncertainty of audiences toward the endorser, and the more likely the receiver develops intimacy toward the endorser.

Apart from the theoretical support, previous studies also provided empirical evidence. Yuan et al. (2016) showed how the credibility of sports stars may influence the PSR of consumers with an endorsed sports brand. Zhang et al. (2020) found that the three sub-dimensions of credibility significantly influence the PSR of receivers with a celebrity. In the social media context, Yılmazdoğan et al. (2021) proved the same result. Based on the above theoretical perspective and empirical evidence,

H2: The credibility of the endorser has a positive impact on the para-social relationship of potential tourists with the endorser.

#### Endorser Credibility and Destination Source Credibility

Trust transfer theory (TTT) (Strub and Priest, 1976) postulated that an individual (i.e., trustor) trust in one source may be transferred to other difference unknown but related sources through communication and cognitive processes (Strub and Priest, 1976; Stewart, 2003). This theory argues that an entity could provide to a trustor a definition of the other unknown target as trustworthy and the trustor accepts or rejects its definition on the basis of his trust for the judgment of this entity (Strub and Priest, 1976; Stewart, 2003). The trust transfer process relies on the unknown entity being perceived by a trustor as having a relationship with the trusted entity. This relation between trusted and unknown entities is based on the perception of similarity, proximity or common fate of a trustor between these entities (Campbell, 1958; Stewart, 2003). As mentioned above, credibility refers to the believability of information and/or its source. From the TTT perspective, the destination endorser is highly related to the endorsed destination. Therefore, the credibility of the destination endorser perceived by potential tourists (i.e., trustors) could be transferred to the endorsed destination source credibility. Therefore, the higher the credibility of the destination endorser, the higher the destination source credibility.

Some empirical studies have tested the trust transfer mechanism. In the context of electronic commerce, Chen et al. (2015) verified that the trust of consumers in electronic commercial platforms positively influences their trust in the seller. Similarly, Xiao et al. (2018) found that the trust of consumers in the Internet has a positive influence on their trust in O2O platforms, which in turn has a positive effect on their trust in merchants. In tourism research, few studies explored the trust transfer mechanism. Lee et al. (2014) verified the mechanism of the attitude of attendants toward the influence of Mega events on their attitude toward the hosting destination from the TTT perspective. Based on the above theoretical perspective and empirical evidence,

H3: The credibility of the destination endorser has a positive influence on destination source credibility.

#### Destination Attitude and Place Attachment

Reitsamer et al. (2016) argued that the concept of destination attachment reflects the perceived value and identification of the destination of tourists and proposed that the cognitive evaluation of tourists of the destination provides the potential of changing their attachment orientation. Their study results showed that the attitude of tourists toward the destination positively influences their place attachment. Prayag et al. (2018) believed that the attitude was an antecedent to place attachment because the attitude toward an object could elicit positive feelings toward it. The finding verified their hypothesis. Specifically, from the potential tourist perspective, Chen et al. (2019) found that the attitude of potential tourists toward a destination positively influences their place attachment. Therefore, we propose that

H4: The attitude of tourists toward the endorsed destination positively influences their place attachment.

#### Para-Social Relationship and Place Attachment

Farnum et al. (2005) proposed that the interaction between the individual and the destination is stronger on the psychological level than the physical level, which implies the role of secondary source on the development of place attachment (Beckley et al., 2007). From the transportation theory perspective (Green and Brock, 2000), the attachment of audiences to media persona is critical to their narrative-based belief change. For destination marketing, Hosany et al. (2020) argued that media persona acts as meaning informants through which the audience could develop attachment toward media settings (e.g., destination) through the para-social interaction with media persona. Prior empirical research found that the celebrity involvement of audiences positively influences their place attachment (Chen, 2018). Similarly, Wong and Lai (2015) proved that the positive effect of the celebrity attachment of audiences on their attachment toward the film destination. Recently, Zhang et al. (2020) found that the PSR has a positive influence on the destination brand love of both potential and previous tourists. Therefore, we propose that

H5: The PSR of potential tourists with the destination endorser may positively influence their place attachment.

#### Destination Source Credibility and Place Attachment

Veasna et al. (2013) investigated the relationship between destination source credibility and the place attachment of tourists. Based on self-congruity theory, the study argued that destination source credibility plays a critical role in building the feelings of tourists toward a destination. Given that destination attachment reflects the positive feelings of tourists and emotional connection with a destination, Veasna et al. (2013) believed that tourists could be attached to a reliable and credible destination. The results showed that destination source credibility positively affects the place attachment of tourists. Tsai (2012) showed that the higher the trust of tourists in the destination, the higher place dependence and affective attachment they have toward the destination. Similarly, Su et al. (2018) found that the destination reputation has a positive influence on the place attachment of tourists. Based on prior empirical findings, we propose that

H6: The destination source credibility may positively influence place attachment.

**FIGURE 1:**
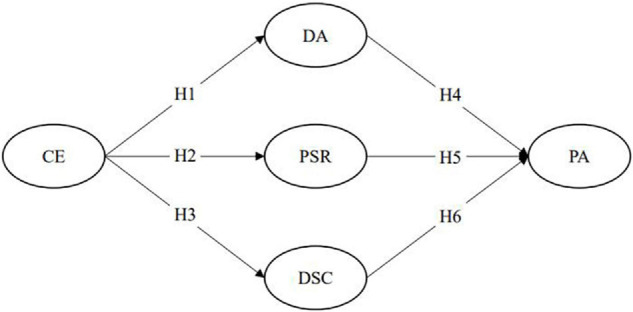
CE = destination endorser credibility; DA = destination attitude; PSR = para-social relationship; DSC = destination source credibility; PA = place attachment.

Based on the theoretical perspectives and prior empirical findings, the corresponding hypotheses are proposed, and Figure 1 presents the research model.

## Research Methods

### Destination Endorser Selection

The Chinese ministry of culture and tourism has recognized the role of social media influencers on rural tourism development (Jiao, 2020). Tenzin becomes one of the most famous destination endorsers in China, when his handsome and innocent smile in his first short video published on the Douyin platform in 2020 left a good impression on audiences (Zhang, 2020; Zhao, 2020). As of March 18, 2021, his social media account such as Sina Weibo, one of the most popular social media platforms in China (CNNIC, 2020), has over 1.68 million^1^ followers. According to the statistics of the Ctrip and the Qunar, two of the Chinese travel platforms, the number of searches for the endorsed destination (i.e., LiTang) and hotel booking shot up rapidly after his first video was released (Leng, 2020; Zhao, 2020). The Chinese ministry of culture and tourism has recognized the “Tenzin effect” on tourism recovery (Guan, 2020). A local state-owned corporation now hires Tenzin to endorse local tourism (Zhao, 2020). Considering that Tenzin has compelling public fame and is the local resident of the endorsed destination, it could facilitate a more favorable audience response (Van der Veen and Song, 2014; Zhang et al., 2020; Roy et al., 2021). Therefore, Tenzin was chosen as the case.

### Measurement Scales and Questionnaire

The measurement scales in the current research were adapted from previous research to conduct a questionnaire survey with Chinese respondents. Prior studies have verified the reliability and validity of the scales. The items were translated following the back-to-back translation process to ensure accuracy. A pilot test with a small number of participants was conducted to modify the item slightly for appropriate wording. The questionnaire includes three parts. In the first part, five screening questions were set to identify subjects. The five questions are as follows: (1) Are you a resident of LiTang? (Samples of residents did not include in this study). (2) Do you know Tenzin? (a photo of Tenzin was provided in the questionnaire to assist respondents in their process of remembering). (3) Do you know that Tenzin is the destination endorser of LiTang? (4) Have you traveled to LiTang before? (5) The respondents over 18 years old were chosen as targets. The second part consisted of 35 items to measure the constructs. The 7-point Likert-type scale was adapted to rate each item. The third part collected the demographic information of respondents. The scale of each construct is described below.

The destination endorser credibility includes three sub-dimensions: trustworthiness, attractiveness, and expertise (Ohanian, 1990). The measurement scale was adapted from Munnukka et al. (2016) as well as Zhang et al. (2020) including four items for trustworthiness, four items for attractiveness, and four items for expertise. The destination attitude was measured using three items (Chen et al., 2019). The para-social relationship was measured using eight items adapted from Reinikainen et al. (2020) and Zhang et al. (2020). The destination source credibility was measured using five items adapted from Veasna et al. (2013) and Girish et al. (2020). Place attachment was measured using two sub-dimensions including four items for place dependence and three items for place identity adapted from Chen et al. (2019) and Hosany et al. (2020).

### Data Collection and Sample Profile

Considering that the destination endorser, Tenzin, is a social media celebrity and conveys information *via* social media platforms, the target subjects in this study are social media user. To collect the samples of targets, an online survey was adopted. This study used two ways to distribute questionnaires. On one hand, the online questionnaire was designed and posted on the Tencent questionnaire platform (an online survey platform)^2^ to collect samples. This online survey platform could automatically screen the questionnaire based on the rate pattern (i.e., those with the same rate number of each item) and response time (i.e., those below the minimal response time), which could assist researchers to ensure the quality of samples. To enhance the response rate, each questionnaire has a 2.5 CNY cash bonus as an incentive. On the other hand, the authors distributed online questionnaires *via* personal social media contacts. The questionnaire was initially sent to the personal network of the author on WeChat; then, the respondents could further send it to their friends, classmates, or colleagues. The samples were collected from March 29 to April 10, 2021. A total of 504 questionnaires were collected and based on the screening questions, 367 questionnaires were usable.

As shown in Table 1, the female (55%) are more than male respondents (45%) in the samples, which shall be acceptable in the context of digital celebrities (Hwang and Zhang, 2018). Most of the respondents were not married, aged 18–35, hold an undergraduate degree, and were working or studying.

**TABLE 1:** Sample profile.

Variable		Frequency (*n* = 367)	%
Gender	Female	202	55
	Male	165	45
Marital Status	Married	125	34.1
	Other	242	65.9
Age	18–25	127	34.6
	26–35	148	40.3
	36–45	79	21.5
	46–55	9	2.5
	56 or above	4	1.1
Education	High school or below	48	13.1
	Undergraduates	235	64.1
	Graduates or above	84	22.8
Occupation	Working	140	38.2
	Student	140	38.2
	Housewife	21	5.7
	Retired	3	0.8
	Others	63	17.1

## Results

The structural equation modeling (SEM) analysis was adopted to verify the hypotheses including two steps: validating the measurement model and path analysis (Anderson and Gerbing, 1988). Compared with PLS-SEM which tends to identify key “driver” constructs, the CB-SEM is more appropriate for theory confirmation (Hair et al., 2016). Therefore, the CB-SEM analysis was adopted, and we process the analysis using the SPSS 25 and Amos 24 software.

### Measurement Model

The first step of SEM aims to verify the reliability and validity of the measurement scales through confirmatory factor analysis (CFA). The results showed that all the indices are acceptable. The indices of model fit showed acceptable results (χ∧2/df = 2.530 < 3, TLI = 0.949 > 0.8, CFI = 0.955 > 0.8, NFI = 0.928 > 0.8, IFI = 0.955 > 0.8, RMSEA = 0.065 < 0.08, SRMR = 0.0454 < 0.08). As illustrated in Table 2, the normality assessment of indicators showed that skewness ranged from −0.82 to 0.094, and the kurtosis ranged from −0.793 to 0.708 within the −1 and 1 acceptable range (Hair et al., 2010). The factor loading of each indicator is above 0.701. The Cronbach’s alpha value of each construct is above 0.7, indicating desirable internal consistency. The composite reliability (CR) and average variance extracted (AVE) are above the minimal values of 0.7 and 0.5, respectively. This finding means that the reliability and convergent validity are acceptable. The Fornell-Larcker criterion (Fornell and Larcker, 1981) was adopted to verify the discriminant validity. As shown in Table 3, the square root of the AVE of each construct is above the correlation of constructs. Therefore, the measurement model has desirable reliability and validity.

**TABLE 2:** Reliability, convergent validity, and normality assessment.

Items	Factor loadings	Cronbach’s alpha	CR	AVE	skewness	kurtosis
**Destination endorser credibility - trustworthiness**						
I feel Tenzin was honest	0.930	0.969	0.969	0.887	–0.772	0.708
I consider Tenzin trustworthy	0.945				–0.693	0.474
I feel Tenzin was truthful	0.952				–0.718	0.441
I consider Tenzin earnest	0.940				–0.820	0.618
**Destination endorser credibility - attractiveness**						
I consider Tenzin very attractive	0.933	0.958	0.958	0.852	–0.321	–0.405
I consider Tenzin very stylish	0.910				–0.119	–0.581
I think Tenzin is good looking	0.909				–0.301	–0.590
I think Tenzin is sexy	0.940				–0.280	–0.646
**Destination endorser credibility - expertise**						
I feel Tenzin knows a lot about the travel destination	0.828	0.938	0.939	0.795	–0.471	0.077
I feel Tenzin is competent to make assertions about the travel destination	0.917				–0.228	–0.275
I consider Tenzin an expert on the travel destination	0.903				–0.118	–0.111
I consider Tenzin sufficiently experienced to make assertions about the travel destination	0.915				–0.353	–0.177
**Destination attitude**						
Attitude toward the destination:						
Very bad- very good	0.940	0.918	0.923	0.802	–0.360	–0.109
Very unfavorable – very favorable	0.950				–0.359	–0.164
Very negative – very positive	0.787				–0.601	0.448
**Para-social relationship**						
I look forward to watching Tenzin on his channel	0.875	0.968	0.969	0.794	–0.315	–0.475
If Tenzin appeared on another program, I would watch that video	0.894				–0.102	–0.658
When I’m watching Tenzin, I feel as if I am part of his group	0.913				–0.045	–0.793
I think Tenzin is like an old friend	0.910				0.094	–0.631
I would like to meet Tenzin in person	0.899				0.094	–0.792
If there were a story about Tenzin in a newspaper or magazine, I would read it	0.879				–0.320	–0.532
Tenzin makes me feel comfortable as if I am with friends	0.924				–0.152	–0.654
When Tenzin shows me how he feels about the destination, it helps me make up my own mind about the destination	0.830				–0.584	–0.235
**Destination source credibility**						
Information claims from the destination are believable.	0.934	0.975	0.975	0.886	–0.370	0.031
The destination is committed to delivering on its claims.	0.931				–0.530	0.044
The destination has a name you can trust.	0.950				–0.359	–0.134
The destination has the ability to deliver what it promises.	0.948				–0.424	–0.026
The destination would deliver what it promises.	0.944				–0.410	–0.085
**Place attachment – place identity**						
I am very attached to the destination	0.830	0.927	0.932	0.821	–0.058	–0.215
This place is very special to me.	0.943				–0.110	–0.555
The place means a great deal to me.	0.940				0.067	–0.403
**Place attachment – place dependence**						
I wouldn’t substitute any other place for the type of experience I can have in the destination	0.905	0.971	0.972	0.896	–0.142	–0.545
I enjoy visiting the destination more than any other place	0.960				–0.070	–0.472
Visiting the destination is more important than visiting any other place	0.964				–0.037	–0.621
I can get more satisfaction out of visiting the destination than from visiting any other place	0.956				–0.097	–0.517

**TABLE 3:** Fornell-Larcker criterion.

	CE-TR	CE-AT	CE-EX	DA	PSR	DSC	PA-PI	PA-PD
CE-TR	0.942							
CE-AT	0.748	0.923						
CE-EX	0.661	0.714	0.892					
DA	0.723	0.643	0.646	0.896				
PSR	0.712	0.797	0.710	0.614	0.891			
DSC	0.709	0.692	0.694	0.726	0.762	0.941		
PA-PI	0.584	0.669	0.670	0.621	0.760	0.688	0.906	
PA-PD	0.601	0.677	0.671	0.616	0.795	0.743	0.769	0.947

*CE-TR = credibility – trustworthiness; CE-AT = credibility – attractiveness; CE-EX = credibility – expertise; DA = destination attitude; PSR = para-social relationship; DSC = destination source credibility; PA-PI = place attachment - place identity; PA-PD = place attachment - place dependence.*

### Structural Model

The second step was to conduct the path analysis to test the hypotheses. The maximum likelihood method was adopted to estimate the parameters of the structural model. The model fit indices are generally accepted (χ∧2/df = 2.609 < 3, TLI = 0.947 > 0.8, CFI = 0.951 > 0.8, NFI = 0.923 > 0.8, IFI = 0.951 > 0.8, RMSEA = 0.066 < 0.08, SRMR = 0.0524 < 0.08). As illustrated in Table 4, the credibility of endorsers has a significant positive influence on the destination attitude of potential tourists (β = 0.785, *t*-value = 15.918, *P* < 0.001), para-social relationship with him (β = 0.871, *t*-value = 17.364, *P* < 0.001) and destination source credibility (β = 0.853, *t*-value = 18.023, *P* < 0.001). Therefore, hypotheses 1, 2, and 3 were supported. Table 4 also reveals that the place attachment of potential tourists is significantly influenced by their destination attitude (β = 0.139, *t*-value = 2.938, *P* = 0.003 < 0.01), para-social relationship with endorser (β = 0.602, *t*-value = 10.874, *P* < 0.001) and destination source credibility (β = 0.262, *t*-value = 4.576, *P* < 0.001). Therefore, hypotheses 4, 5, and 6 were supported.

**TABLE 4:** Hypotheses test.

Hypotheses			β	*t*-value	*P*
Endorser credibility	⇒	Destination attitude	0.785	15.918	< 0.001
Endorser credibility	⇒	Para-social relationship	0.871	17.364	< 0.001
Endorser credibility	⇒	Destination source credibility	0.853	18.023	< 0.001
Destination attitude	⇒	Place attachment	0.139	2.938	< 0.01
Para-social relationship	⇒	Place attachment	0.602	10.874	< 0.001
Destination source credibility	⇒	Place attachment	0.262	4.576	< 0.001

The mediating effect of the credibility of endorsers on the place attachment potential tourists was verified *via* the bootstrap method (Kline, 2015). The number of bootstrap samples is set as 5,000 (Hair et al., 2016), and the bias-corrected confidence level is 95%. The lower bounds (BC) and upper bounds (BC) were 0.816 and 0.889, respectively, excluding zero in this range. The result showed that a significant indirect effect exists between endorser credibility and place attachment (*P* < 0.001). The total indirect effect of endorser credibility is 0.857 which separately influences through destination attitude (γ = 0.109), para-social relationship (γ = 0.524), and destination source credibility (γ = 0.224).

## Discussion and Conclusion

### Theoretical Implications

Although the persuasiveness of destination endorsers in destination marketing has been widely identified (van der Veen, 2008; Glover, 2009; McCartney and Pinto, 2014; Van der Veen and Song, 2014; Xu and Pratt, 2018), few studies explored their influence mechanism on the place attachment of potential tourists which could improve the destination competitiveness (Dwyer et al., 2019). By adopting a quantitative research method, this study verified the advantage of a credible destination endorser in place attachment formation to extend the tourism and marketing literature.

The current study tested the relationship between destination endorser credibility and destination attitude from the source credibility model perspective in the marketing field. Consistent with prior findings of McCartney and Pinto (2014), the result supports this hypothesis which implies that a credible destination endorser may positively change the attitude of potential tourists toward the destination through the internalization process.

From the uncertainty reduction theory perspective in the social-psychological field, this study established and verified the influence relationship between credibility from the marketing field and para-social relationship from the media psychology field. The finding of this study is consistent with the findings of prior studies (Yuan et al., 2016; Zhang et al., 2020; Yılmazdoğan et al., 2021). Moreover, this study further clarifies the underlying reason of this relationship, which indicated that a credible endorser could reduce the uncertainty of potential tourists which would facilitate the potential tourists building a para-social relationship with the endorser.

Trust transfer theory from the psychology field has rarely been introduced in the tourist marketing literature to explain the endorser credibility influence (Lee et al., 2014). The current study revealed that the credibility of endorsers positively influences destination source credibility, which is consistent with prior studies (Lee et al., 2014; Chen et al., 2015; Xiao et al., 2018). The finding suggests that a credible endorser could provide a definition of a destination where an individual has not visited before as trustworthy. Considering the lack of studies exploring this relationship in tourism marketing literature, further studies could verify it in other different contexts.

Consistent with previous empirical findings (Tsai, 2012; Veasna et al., 2013; Wong and Lai, 2015; Reitsamer et al., 2016; Chen, 2018; Prayag et al., 2018; Chen et al., 2019), our study verified the relationship among destination attitude, para-social relationship, destination source credibility, and the place attachment of potential tourists. The bootstrap method verified the indirect effect of destination endorser credibility on place attachment through the above three constructs. Therefore, this study extends the tourism destination marketing literature by verifying the three different influence mechanisms of endorser credibility on pre-visit place attachment and increasing the insight on how to enhance the behavior intention and loyalty of tourists toward a destination.

### Practical Implications

Understanding the behavior of tourists is important for destination managers (Vu et al., 2020). Given the effective role of place attachment on the behavior intention and destination loyalty of tourists, this study highlights the advantage of a credible destination endorser in pre-visit place attachment formation by identifying three possible influence mechanisms which would provide destination managers with several practical implications.

Firstly, this study reveals that destination managers could gain a competitive advantage through forming the place attachment of potential tourists. Since potential tourists do not experience the destination before, therefore, this study highlights the role of destination endorser. The credibility of endorsers could foster potential tourists through a change in their destination attitude. For example, the endorser could convey the desirable image, uniqueness, value, characteristics, or lifestyle embodied in their destination to target potential tourists through social media content such as vlog. Thereafter, the credibility of endorsers would facilitate the internalization process of potential tourists of the information conveyed, which in turn changes their attitude and the orientation of place attachment (Reitsamer et al., 2016).

Secondly, our study reveals that a credible destination endorser could facilitate the para-social interaction of potential tourists. Therefore, destination managers should select a credible endorser, enlarge their communication channels such as various social media platforms and enhance the interaction between endorser and destination. For example, the endorser should pay attention to their interaction with tourists in his/her media channel such as live broadcast, provide more interactive games or activities to enhance the involvement of tourists. Thereafter, the credible endorser could enhance the possibility that a potential tourist fosters a para-social relationship with him/her, which in turn forms the place attachment of potential tourists (Hosany et al., 2020).

Thirdly, in an information explosion era and information asymmetric context, gaining the trust of potential tourists is important (Erdem and Swait, 2004). The result indicates that a credible destination endorser positively influences the destination source credibility. To build the connection between endorsers and tourists, more daily information relevant to endorsers and destinations should provide through media channels to make tourists more familiar with the endorser. The endorser should pay attention to their information openness and show his/her credibility characteristics to gain the trust of tourists. Thereafter, the destination credibility would build the feeling of potential tourists toward it, which in turn enhances their place attachment (Veasna et al., 2013) before travel.

## Conclusion and Limitations

In conclusion, this study extends the literature of forming the place attachment of potential tourists from the personal characteristics perspective of the destination endorser. The findings highlight the influence mechanism of the credibility of endorsers on the place attachment of potential tourists through the mediating role of the destination attitude, destination credibility, and para-social relationships of tourists. We hope that this study can inspire further research in this field.

Although this study extends the tourism marketing literature by exploring the influence of the credibility of the endorser on forming pre-visit place attachment, some limitations remain and require consideration when interpreting the findings. Firstly, whilst the various theoretical foundations are introduced in our research model, the cross-sectional study may influence the causal inferences. Secondly, the destination endorser selected in this study is a local resident of the endorsed destination. Therefore, the results may not be generalizable to the non-local resident study case. Thirdly, the female and male percentage in the sample is slightly uneven. The underlying reason may be that the endorser chosen in this study is male. Fourthly, since the sample mostly includes Generation Z and Y respondents, those respondents are seeming more familiar with social media and influencers, which may also influence the results such as trust transfer mechanism or para-social relationship formation.

Future studies could explore the difference in the influence mechanism between local endorsers and non-local endorsers. In addition, further studies could identify other influence mechanisms between endorser credibility and place attachment. The influence of the attributes and personalities of other endorsers on place attachment should also be explored in the future.

## Data Availability Statement

The raw data supporting the conclusions of this article will be made available by the authors, without undue reservation.

## Author Contributions

ZS collected the data and drafted the manuscript. JL directed the manuscript writing and revised it. Both authors jointly contributed to the development of the research framework, contributed to the article, and approved the submitted version.

## Conflict of Interest

The authors declare that the research was conducted in the absence of any commercial or financial relationships that could be construed as a potential conflict of interest.

## Publisher’s Note

All claims expressed in this article are solely those of the authors and do not necessarily represent those of their affiliated organizations, or those of the publisher, the editors and the reviewers. Any product that may be evaluated in this article, or claim that may be made by its manufacturer, is not guaranteed or endorsed by the publisher.

## Footnotes

1
https://www.weibo.com/u/7524392184

2
https://wj.qq.com/
